# Extracellular Vesicles from Different Mesenchymal Stem Cell Types Exhibit Distinctive Surface Protein Profiling and Molecular Characteristics: A Comparative Analysis

**DOI:** 10.3390/ijms26073393

**Published:** 2025-04-04

**Authors:** Atziri G. Fernández-Pérez, Azucena Herrera-González, Edgar J. López-Naranjo, Iliany Annel Martínez-Álvarez, David Uribe-Rodríguez, Daniel E. Ramírez-Arreola, María Judith Sánchez-Peña, Jose Navarro-Partida

**Affiliations:** 1 Centro Universitario de Ciencias Exactas e Ingenierías (CUCEI), University of Guadalajara, Guadalajara 44430, Jalisco, Mexico; atziri.fernandez@alumnos.udg.mx (A.G.F.-P.); mariaa.herrera@academicos.udg.mx (A.H.-G.); edgar.lopezn@academicos.udg.mx (E.J.L.-N.); maria.spena@academicos.udg.mx (M.J.S.-P.); 2 Centro de Biotecnología Santer S.C., Guadalajara 45040, Jalisco, Mexico; ilianyannel@gmail.com (I.A.M.-Á.); daviruscell@gmail.com (D.U.-R.); 3 Centro Universitario de la Costa Sur (CUCSUR), University of Guadalajara, Autlan 48900, Jalisco, Mexico; eden.ramirez@academicos.udg.mx; 4 School of Medicine and Health Sciences, Monterrey Institute of Technology, Zapopan 45201, Jalisco, Mexico

**Keywords:** extracellular vesicles (EVs), EVs of human placenta-derived mesenchymal stem cells (hP-MSC-EVs), EVs of human endometrium-derived mesenchymal stem cells (hE-MSC-EVs), EVs of human dental pulp-derived mesenchymal stem cells (hDP-MSC-EVs)

## Abstract

The current medical need to respond to different diseases has sparked great interest in extracellular vesicles (EVs) derived from mesenchymal stem cells (MSCs) due to their great regenerative potential and as drug carriers by playing a critical role in cell–cell communication. However, due to their heterogeneity, there is no standardized universal method for their identification and characterization, which limits their clinical application. This study, following the recommendations and methodologies proposed by MISEV2023 for the characterization of EVs, shows for the first time a detailed morphological, protein, and biochemical comparison between EVs derived from three different MSCs sources (placenta, endometrium, and dental pulp). The information obtained from the different applied assays suggests that there are substantial differences between one EVs source and another. It also offers valuable insights that provide the guidelines to ease their profiling and therefore improve their selection, in order to speed up their use and clinical application; additionally, the knowledge obtained from each characterization test could facilitate new researchers in the field to choose a specific cell source to obtain EVs and select the appropriate methods that provide the necessary information according to their requirements.

## 1. Introduction

Extracellular vesicles (EVs) are nano-sized, heterogeneous, and lipid bilayer particles that contain different molecular compositions regarding proteins, nucleic acids, and phospholipids from their origin cell, and cannot replicate on their own [1,2,3]. Almost all cells of all living organisms on Earth shed EVs into the extracellular milieu [4]. In addition to their molecular composition, the size and biogenesis of EVs are factors that can distinguish them from each other.

Communication between cells and their environment is an important process in living organisms; EVs play diverse and crucial roles in pathophysiological processes and the acellular (non-living) mode of cell–cell communication [5]. In addition, their low immunogenicity, biocompatibility, ability to cross biological barriers, secretion, and easy recovery from all body fluids have made them stand-out tools for diagnostic and therapeutic purposes [4,6].

**Figure 1:**
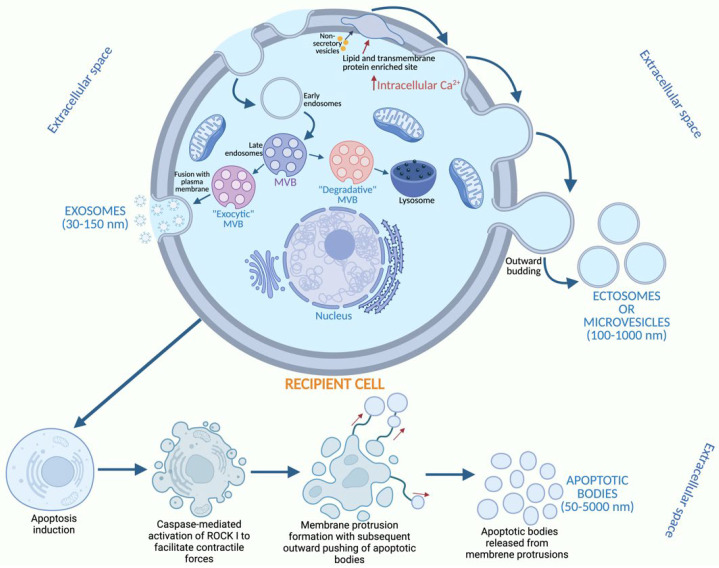
Biogenesis and release of extracellular vesicles. Exosomes are formed by multivesicular bodies (MVBs). Ectosomes are formed by the plasma membrane, and apoptotic bodies by disassembly of an apoptotic cell or programmed cell death (drawn using the BioRender.com).

Based on their biogenesis, EVs can be classified into three subtypes: exosomes, microvesicles (MVs) or ectosomes, and apoptotic bodies [7] (Figure 1). Exosomes are vesicles formed inside multivesicular bodies (MVBs) within cells. When there is a process of endocytosis in a cell, this results in the formation of early endosomes which are transformed into late endosomes, which later give rise to MVBs. These MVBs contain components of the extracellular milieu like lipids, small molecules, metabolites, and proteins. MVBs can follow two pathways. In the first, MVBs can fuse and degrade their contents with lysosomes, without releasing exosomes. In the second, MVBs can take the exocytosis pathway and fuse with the plasma membrane, and then release their content into the extracellular space. Once MVBs content is released into the extracellular space, exosomes are liberated [8,9]. On the other hand, ectosomes come from the plasma membrane, through outward budding, due to the gradual loss of the interaction between the cytoskeletal proteins and the plasma membrane. This is due to the increase in protein degradation and cytosolic calcium that can induce the disassembly of the cytoskeleton, giving rise to budding formation. This budding is favored by the activation of the lipid translocases which prompt changes in the lipid bilayer, giving rise to the release of ectosomes [10]. Lastly, apoptotic bodies, which are mere debris of the cell, are formed exclusively during the disassembly of an apoptotic cell or programmed cell death. Depending on the cell type and mechanism used by them for cell disassembly, different qualities and quantities of apoptotic bodies will be generated. Some cells can form preclusive structures, which are released from membrane protrusions into the cellular space, while others can use complex processes following coordinated morphological steps [10,11].

Each EVs can differ in its cargo composition and molecular characteristics, but in general, there are a lot of investigations that coincide in some specific features, like their size, density, biogenesis, and enriched proteins (Table 1).

To date, several different cells have been studied as sources of EVs release, but mesenchymal stem cells (MSCs) have been shown to have great therapeutic potential compared to other sources. MSCs are multipotent adult stem cells that can be easily derived from a variety of human tissues. MSCs have been extensively evaluated as a potential therapeutic option due to their biological properties, such as their capacity to modulate immune responses, their low immunogenicity, and their ability to regenerate damaged tissues, all of which have been demonstrated in the treatment of various diseases [19,20]. Furthermore, MSCs can be obtained from ethically acceptable sources such as bone marrow aspirates and adipose tissue, which facilitates their procurement [21,22]. Accumulating evidence suggests that the primary therapeutic benefits of MSCs in tissue repair and inflammatory diseases are predominantly mediated by the MSC-derived products they secrete (i.e., the “secretome”), rather than by the MSCs themselves. The “secretome” of MSCs can be divided into two primary constituents: a soluble component consisting of secretory proteins such as growth factors, cytokines, and other metabolites, and a vesicular component composed predominantly of EVs, which can deliver their entire contents to receptor cells via paracrine and endocrine signaling to regulate their function. This “secretome” has been shown to induce many of the therapeutic properties associated with MSCs [23,24,25].

Several MSC-based therapies have been approved in different countries (e.g., Prochymal^®^ and CARTISTEM^®^) [26,27]. So far, various clinical trials have been registered to test them on diverse medical conditions including cardiovascular, neurological, respiratory, hematological/oncological malignancies, and immune-deficient or inflammatory conditions [28,29,30,31,32].

MSCs can be derived from a variety of tissues including umbilical cord blood, menstrual blood, placenta, dental pulp, skeletal muscle, skin, and Wharton’s jelly [5,20,25,32,33,34,35,36,37]. These MSCs exhibit different functional phenotypes depending on their tissue origin. Interestingly, side-by-side comparisons have revealed significant differences between MSCs populations from different sources, suggesting that the tissue of origin may influence the characteristics and therapeutic potential of these cells.

MSC-derived EVs (MSC-EVs) have been observed to have multiple therapeutic benefits (Table 2). When tested in various disease models, MSC-EVs of different origins have shown similar or even superior therapeutic capacity to their parental cells. In addition, MSC-EVs have safety advantages as they are considered non-immunogenic and carry a lower risk of inducing allogeneic immune rejection by the host.

Although much research has been performed on the characteristics and properties of MSC-EVs, it remains unclear how this might contribute to their selection for therapeutic use. To try to resolve these doubts, this comprehensive study characterized and compared EVs derived from three human MSCs types (EVs of human placenta-derived MSCs (hP-MSC-EVs), EVs of human endometrium-derived MSCs (hE-MSC-EVs), and EVs of human dental pulp-derived MSCs (hDP-MSC-EVs)). Several analytical techniques were used to characterize MSC-EVs (dynamic light scattering (DLS), transmission electron microscopy (TEM), scanning electron microscopy (SEM), MACSPlex surface protein profiling, Fourier-transform infrared spectroscopy (FTIR), and Raman spectroscopy). Thus, this research significantly contributes to a better understanding of the morphological, proteomic, and chemical characteristics of three different sources of MSC-EVs and how these might influence their selection for use in specific clinical applications.

## 2. Results

### 2.1. Characterization of MSC-EVs

**Figure 2:**
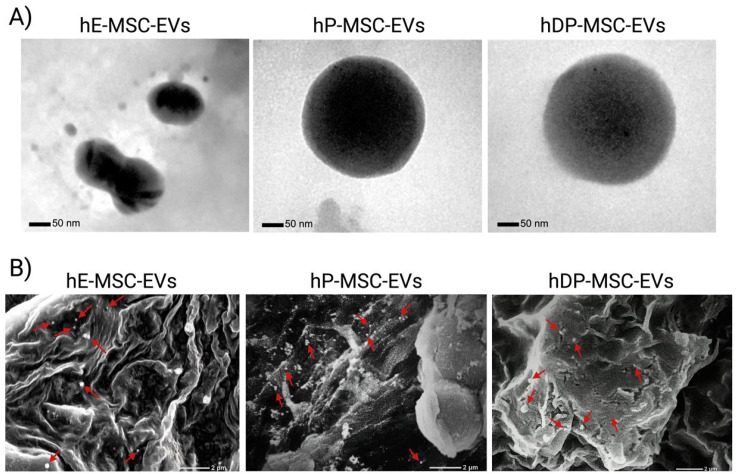
Electronic micrographs of extracellular vesicles derived from mesenchymal stem cells (MSC-EVs). (**A**) Morphological assessment of MSC-EVs at the single-vesicle level by transmission electron microscopy; scale bar: 50 nm. (**B**) Presence and morphological assessment of MSC-EVs by scanning electron microscopy; scale bar: 2 μm (EVs are marked with red arrows to facilitate their observation). Abbreviations: hE-MSC-EVs, EVs of human endometrium-derived mesenchymal stem cells; hP-MSC-EVs, EVs of human placenta-derived mesenchymal stem cells; hDP-MSC-EVs, EVs of human dental pulp-derived mesenchymal stem cells.

**Figure 3:**
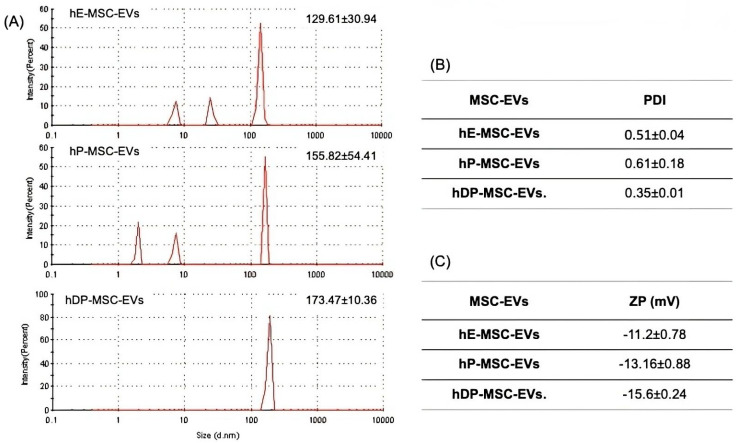
Dynamic light scattering. (**A**) Representative size distribution profile assessment of MSC-EVs obtained by the average diameter and the standard deviation of each sample. (**B**) The average polydispersity index (PDI) of MSC-EVs, and the standard deviation of each sample. (**C**) The zeta potential measurement of MSC-EVs, the average of the net negative charge, and the standard deviation of each sample. Note: the measures of size distribution, PDI, and zeta potential were calculated by the consecutive run of three representative samples. Abbreviations: hP-MSC-EVs, EVs of human placenta-derived mesenchymal stem cells; hE-MSC-EVs, EVs of human endometrium-derived mesenchymal stem cells; hDP-MSC-EVs, EVs of human dental pulp-derived mesenchymal stem cells.

EVs were isolated from three different human mesenchymal stem cell sources (i.e., endometrium, placenta, and dental pulp) by a typical differential ultracentrifugation method. TEM images (Figure 2A) showed the morphology of the hE-MSC-EVs, which showed slightly oval edges, and the round and defined morphology of hP-MSC-EVs and hDP-MSC-EVs. On the other hand, the SEM micrographs (Figure 2B) were taken at a scale of 2 μm. The EVs are indicated by red arrows to specify their location due to the scale used. Other structures could also be observed, possibly protein aggregates and cellular debris. DLS was performed to evaluate the size distribution and measure the zeta potential of each MSC-EV subpopulation. Concerning the size range, it was observed that hE-MSC-EVs and hP-MSC-EVs were heterogeneous but within the normal EV size range. On the other hand, the size distribution of hDP-MSC-EVs was homogeneous due to the presence of a single peak in the evaluation of the average diameter. The other subpopulations (hP-MSC-EVs and hE-MSC-EVs) present more than one peak, which in conjunction with the average polydispersity index (PDI) reaffirmed the heterogeneity of the samples (Figure 3A,B). Finally, the zeta potential, a measure of surface charge, showed that EVs carry a net negative charge and fall within the normal range of the EVs, which proved the presence of negatively charged sialic acid on EVs membrane-bound oligosaccharides (Figure 3C).

### 2.2. MACSPlex Surface Protein Profiling

**Figure 4:**
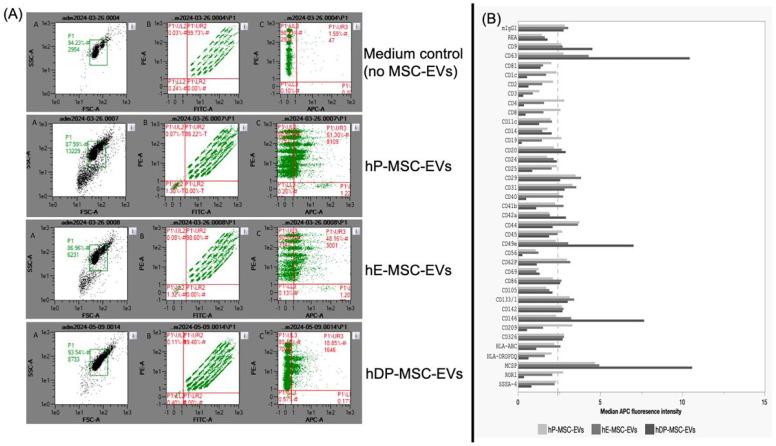
Surface marker profile of extracellular vesicles (EVs) isolated from three different sources of mesenchymal stem cells (MSCs) (dental pulp, placenta, and endometrium), profiled with a multiplex bead-based flow cytometry assay for the detection of EV surface signatures. (**A**) Results after analyzing the extracellular vesicles derived from mesenchymal stem cells (MSC-EVs) compared to the medium control (no MSC-EVs), identifying 39 distinct bead populations based on their fluorescence in the FITC vs. PE channels. Dot plots were used to visualize the APC-stained bead populations. Calibration beads (gate selection by size and granularity), first column. The determination of the target area and the 39 epitope areas is given in the second column, and the third column shows the adherence of MSC-EVs to the beads. (**B**) Graphical representation of the median APC fluorescence intensity detected (background subtracted for each marker analyzed by the MACSPlex EV IO kit for all MSC-EV samples). Abbreviations: hP-MSC-EVs, EVs of human placenta-derived mesenchymal stem cells; hE-MSC-EVs, EVs of human endometrium-derived mesenchymal stem cells; hDP-MSC-EVs, EVs of human dental pulp-derived mesenchymal stem cells.

In this study, we aimed to perform the surface protein profiling of three different MSC-EVs (hP-MSC-EVs, hE-MSC-EVs, and hDP-MSC-EVs) with a multiplex bead-based flow cytometry assay platform for EVs research. This assay comprises 39 hard-dyed capture bead populations, each coated with monoclonal antibodies targeting 37 potential EVs surface epitopes and two internal isotype controls. The assay method ensures that only extracellular vesicles are detected and not free proteins, allowing specific identification of EVs in both cell culture supernatants and biological fluids without additional purification. The signal levels detected correspond directly to the abundance of the respective surface proteins present in the EVs samples. All bead populations were identified and gated based on their respective fluorescence intensity detected in the FITC and PE channels of flow cytometers, according to the assay documentation provided by the manufacturer (MACSPlex EV IO kit, Biotec Ltd., Almac House, UK). After incubating EV-containing samples, the captured EVs were then detected by counterstaining with APC-labeled detection antibodies against the tetraspanins CD9, CD63, and CD81. Figure 4A shows the results obtained by the MACSPlex EV IO kit, which were selected according to the size (FSC) and granularity (SSC) graph, using the positive medium control population (no MSC-EVs), and the populations selected for analysis (hP-MSC-EVs, hE-MSC-EVs, and hDP-MSC-EVs).

The multiplex assay simultaneously includes different kinds of proteins: the tetraspanins CD9, CD63, and CD81, which are often referred to as EV surface markers, and other surface proteins such as cell activation markers (i.e., CD44, platelet activation marker CD62P (P-selectin)), proteins indicating cellular origin (i.e., CD3, CD4, CD8, CD14, CD41b, CD42a, CD45, and CD62P), and antigen-presenting proteins (i.e., HLA-DR/DP/DQ, CD86). In this assay, all proteins were detected above the detection threshold in all MSC-EVs samples at varying intensities. The representation of the quantitative analysis of the median APC fluorescence intensities across all bead populations, with background subtraction, is shown in Figure 4B.

As expected, some widely used EVs markers CD9 and CD63 were identified in MSC-EVs in all measured samples. In addition, some markers of the cell origin (endothelial, epithelial, and MSCs), cell activation, and functional status of EVs (i.e., CD31, CD142, CD29, CD133/1, CD326, and MSCP) were found to be highly expressed on EVs in all sample units, whereas the markers CD3, CD56, CD69, and HLA-DRDPDQ showed low expression on MSC-EVs, as shown in Figure 4B. A more detailed presentation of the results of individual cases showed that only some markers were presented by one type of MSC-EVs. In hP-MSC-EV lineage-associated markers, markers associated with immune and antigen-presenting cells (i.e., CD8, CD19, and ROR1), antigen-presenting proteins (i.e., CD4 and CD209), and the hematopoietic marker CD45 were highly expressed, whereas the lineage-associated markers CD41b and HLA-ABC were more present in hE-MSC-EVs. Finally, the lineage-associated marker CD42a was highly expressed in hDP-MSC-EVs (Figure 4B, Table 3).

In order to provide a better understanding of the results obtained from the protein profile of the MSC-EVs analyzed in this study, Table 3 was generated in a more summarized manner, giving an overview of the functionality of each surface protein marker and the type of MSC-EVs in which it was found.

### 2.3. Fourier-Transform Infrared (FTIR)

**Figure 5:**
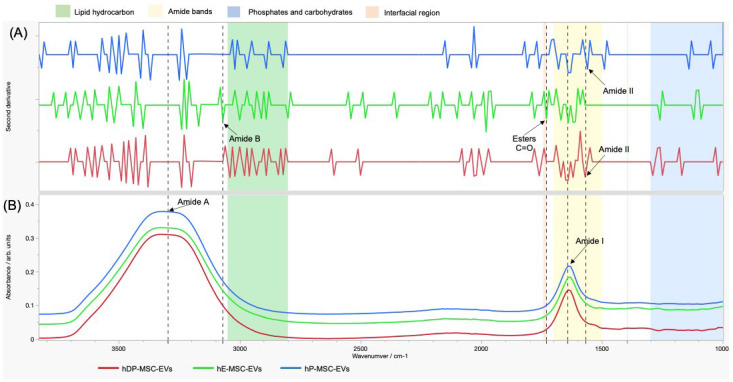
Average FTIR spectra of EVs obtained from three different MSC sources (hP-MSC-EVs, hE-MSC-EVs and hDP-MSC-EVs). (**A**) FTIR spectrum after application of the second derivative, showing the presence of the secondary protein structures, phosphates, carbohydrates, and lipids. (**B**) FTIR spectrum without modification, showing the presence of amides. Abbreviations: hP-MSC-EVs, EVs of human placenta-derived mesenchymal stem cells; hE-MSC-EVs, EVs of human endometrium-derived mesenchymal stem cells; hDP-MSC-EVs, EVs of human dental pulp-derived mesenchymal stem cells.

For molecular characterization of the EVs, an FTIR spectrum average was generated through 50 scans per sample, and analyzed by applying the corresponding assignment to the prominent peaks of the EV spectra (Table 4). The FTIR spectrum in the infrared region suggested the presence of amides (Figure 5B). By applying and analyzing the second derivative of the data, more components could be observed, including lipids, carbohydrates, phosphates, and the secondary structure of the proteins (Figure 5A), allowing a clearer observation of their composition.

### 2.4. Raman Spectroscopy

**Figure 6:**
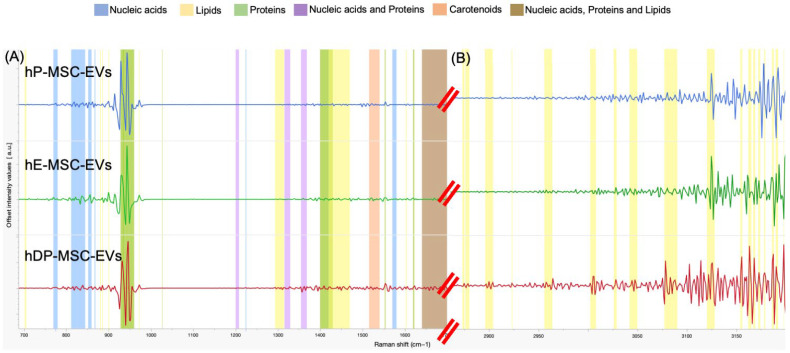
Second derivative of the EVs’ Raman fingerprints in the spectral range (**A**) 700 cm^−1^ to 1700 cm^−1^ (left) and (**B**) 2850 cm^−1^ to 3200 cm^−1^ (right) of hP-MSC-EVs, hE-MSC-EVs, and hDP-MSC-EVs. Average Raman spectra obtained using an excitation wavelength of 785 nm and 25 s for 15 pulsations of exposure for each spectrum. Abbreviations: hP-MSC-EVs, EVs of human placenta-derived mesenchymal stem cells; hE-MSC-EVs, EVs of human endometrium-derived mesenchymal stem cells; hDP-MSC-EVs, EVs of human dental pulp-derived mesenchymal stem cells.

The three sources of MSC-EVs were analyzed by Raman spectroscopy in the spectral ranges of 700–1700 cm^−1^ (left) and 2850–3200 cm^−1^ (right), which are the most significant regions of the Raman spectrum for biological specimens. Figure 6 shows representative mean Raman spectra (±1 standard deviation) of the MSC-EVs. The Raman spectra of the MSC-EVs samples are very similar in lipids, proteins, and nucleic acid band positions (Table 5), differing in the intensity of the signal of each compound.

## 3. Discussion

Owing to their innate origins, biocompatibility, capacity to traverse key biological barriers, and diminished toxicity and immunogenicity, EVs have been employed in diverse therapeutic strategies as drug delivery platforms and as sources of effective therapeutics [15,74]. Given that nearly all cell types release EVs, a lot of different cell sources have been investigated, especially EVs derived from MSCs due to the fact that it has been verified by various scientific groups that MSC-EVs retain the therapeutic properties of the parent cell [75,76,77]. EVs released by MSCs possess numerous advantages, even more than the MSCs themselves, and have promising applications as drug delivery systems [78]. Due to EVs exhibiting significant heterogeneity in size and molecular makeup, their characterization and clinical application become challenging when choosing the best one to use in a specific treatment for a specific disease [15,52].

For EVs to be successfully employed as drug delivery systems, their characterization and efficient drug loading must be well established. In this context, our study offers valuable insights into the molecular characteristics of EVs derived from diverse MSCs subpopulations.

In this comparative study, we performed a comprehensive quantitative analysis of the molecular characteristics of MSC-EVs isolated from three different sources. Our results demonstrate substantial variations in the surface marker profile of EVs obtained via different MSCs sources, as well as in the size, zeta potential, and chemical profile, highlighting the potential implications of these differences for therapeutic applications. We recognized the multifaceted nature of EVs and their diverse characteristics, acknowledging that all these aspects may not fully be enough to understand and elucidate the complexity of their behavior and therapeutic potential. Nonetheless, this comparative report may offer insights into the differential protein content, morphological and physical characteristics of EVs based on the tissues of MSCs employed in their isolation.

MSCs secrete various types of EVs that differ in size, composition, and biological activities [4,16,79]. As shown in Figure 4, Figure 5 and Figure 6, hE-MSC-EVs, hP-MSC-EVs, and hDP-MSC-EVs are three subpopulations with different characteristics, including size, biochemical composition, and marker expression. Concerning the morphology, TEM images showed that all MSC-EVs subpopulations exhibited a round morphology with well-established structures of rounded and ovoid edges, within typical limits. On the other hand, the SEM images demonstrated the existence of vesicles exhibiting a spherical shape and varying dimensions within the EVs preparations. We can also observe the presence of other compounds, which could mean that there are aggregates of proteins and cellular compound debris in the EVs samples. The size range obtained by DLS was 98.67–183.83 nm; specifically, hDP-MSC-EVs showed a larger size (173.47 ± 10.36 nm) and a PDI value of less than 0.4 (0.35 ± 0.01), which is typically associated with a homogeneous population of lipid-based carriers, such as liposomes. On the other hand, the samples of hE-MSC-EVs and hP-MSC-EVs were more heterogeneous, with PDI values of 0.51 ± 0.04 and 0.61 ± 0.18, and with smaller sizes of 129.61 ± 30.94 and 155.82 ± 54.41, respectively, suggesting that within the three different sources of MSC-EVs, there could be exosomes, ectosomes, and apoptotic bodies, based solely on the size of the EVs [7,80,81].

Zeta potential is considered one of the most valuable tools for studying the collective properties and behavior of nanoparticles, including the colloidal stability of EVs in dispersed systems. This method has great potential for studying the activity and role of EVs in biological processes. In addition, surface charge influences various biological processes associated with them, such as cellular uptake and cytotoxicity. In short, the surface charge of extracellular vesicles can be used to assess their adhesion to target substrates, allowing prediction of whether they will attach to surfaces or be internalized by target cells. Thus, zeta potential can be exploited in the design of EV-based therapeutics that rely on electrostatic adsorption [82]. The zeta potential represents the electric potential around the surface of a charged particle, which is influenced, among other things, by the covalent attachment of negatively charged sialic acid molecules to the terminal ends of N- and O-linked glycoproteins and glycosphingolipids. The measured zeta potentials are considered neutral when they are between −10 and +10 mV but are classified as strongly positively or strongly negatively charged when they exceed +30 mV or fall below −30 mV, respectively [83]. On the other hand, zeta potentials ranging from −10 mV to −50 mV (in phosphate-buffered saline (PBS) or dilutions thereof) have been reported for vesicles derived from cell culture or plasma samples. However, there was a decrease in vesicles isolated from the plasma of patients with certain cancers and heart failure. In general, significant changes in zeta potential have been observed in EVs derived from cells of healthy patients compared to patients with some diseases, which have been related to the average circulating concentration of EVs in the patients. On the other hand, it is worth highlighting a relevant point in this difference, which is the fact that the standard deviation of the zeta potential is usually higher in EVs derived from damaged cells [84,85,86]. In this sense, the zeta potential of the EVs studied in this research, which was measured by DLS, can be considered neutral, since the zeta potential values obtained are in the range of −10.42 mV to −15.84 mV, with a minimum standard deviation that does not exceed the unit, which can mean that the progenitor cells from which they originate have not been damaged by any pathology. Therefore, these types of EVs can be used as drug carriers for the treatment of diseases.

Regarding the surface protein profile of EVs, MISEV2023 mentions that to date, no molecular class measurement can quantify all EV markers. Hence, the universality of EVs is not yet fully clear and accepted [3]. The use of tetraspanins CD9, CD63, and CD81 is not specific for the identification of exosomes as a subtype of EVs due to their heterogeneity, so MISEV2023 recommends applying different methods to evaluate the protein content of EVs based on a five-category framework to assess the presence of certain EV characteristics, the purity of common contaminants, and possible intracellular origin of EVs or co-isolates. To analyze the protein profile of the three subtypes of stem cell-derived EVs, a proteomic analysis was performed using a flow cytometry assay (MACSPlex) based on the capture of EVs by capture beads coated with a specific antibody that reacts with an analyte present in the sample and can be identified by its specific fluorescence characteristics. Based on the protein profiling results obtained with the MACSPlex EV IO Kit and the results of previous investigations, using the same MSC sources to obtain EVs, we can elucidate the biological conditions of the MSC-EVs analyzed in this study. The presence of CD44, CD29, CD9, and CD63 with an above-average APC intensity on hP-MSC-EVs was confirmed by Zhang et al. 2020 and Zheng et al. (2023) who used the same type of MSC-EVs, identifying certain biological activities as the ability to reduce liver fibrosis and proangiogenic effects on endothelial cells, enhance angiogenesis, and improve neurological function, which represent a promising strategy for the treatment of spinal cord injury [39,40]. In comparison with the studies of Wang, K. et al. (2017) and Marinaro, F. et al. (2018) on the presence of certain surface markers for hE-MSC-EVs, we also confirmed the presence of CD29, CD44, CD49e, CD9, and CD63, and the absence of CD45 and HLA-DR markers. This confirms the origin of the hE-MSC-EVs used in this assay and therefore the biological activity such as paracrine effect in cardiac repair, antioxidant activity, and the capacity to be adjuvants in in vitro fertilization [43,44]. Finally, our results indicated the presence of CD9 and CD63 and the absence of CD45 on hDP-MSC-EVs, in accordance with Amaro-Prellezo, E. et al. (2024) and He, X. et al. (2025). These studies show that hDP-MSC-EVs modulate biological processes related to inflammation, have cardioprotective properties, promote bone regeneration through osteogenic and osteoinductive effects, and create an environment conducive to cell growth [41,42].

The results obtained with the MACSPlex EV IO Kit and the comparisons between the different studies will allow us to guarantee the functional status and origin of the MSC-EVs analyzed in this study, and to promote their application as adjuvants in the treatment of various diseases, either as drug carriers or directly.

To analyze the molecular composition of EVs, two spectroscopic methods were used: one based on infrared radiation passed through the sample in question (FTIR) and the other based on the interaction of light with the chemical bonds of the matter or substance being studied (Raman). The results obtained by both methods were in agreement since both confirmed the presence of proteins and lipids. The FTIR spectra provided a better characterization of the proteins present in the EVs and their structures, with the two prominent amide-mediated absorption bands observed in the three EVs samples analyzed highlighting the difference concerning the presence of amide B and the ester group in hE-MSC-EVs and the presence of amide II in hP-MSC-EVs and hDP-MSC-EVs, but not in hE-MSC-EVs (Table 4, Figure 5). The two prominent amide absorption bands present in the samples are a key feature of their biological origin. It should be noted that the presence of these components is consistent in all MSC-EVs samples analyzed; however, the protein–lipid ratio based on absolute values provided by FTIR is not absolute but rather reflects differences in concentrations or proportions between one EVs sample and another. Previous studies have observed that the presence of higher phosphate and carbohydrate content is a particular characteristic of microvesicles due to their origin in the plasma membrane. On the other hand, the intensity of the C=O stretch band is suppressed in exosomes. Finally, in apoptotic bodies, the amide stretching bands were observed to be more pronounced. Therefore, the results obtained in this study may suggest that in the hP-MSC-EVs and hDP-MSC-EVs samples, we can find exosomes and apoptotic bodies due to the suppression of the C=O stretch and the prominent amide bands. Regarding hE-MSC-EVs, the suspension could be composed of apoptotic bodies due to the presence of an amide band. It could also be said that exosomes are absent due to the presence of the C=O stretch. Regarding the presence of microvesicles, it can be said that all three MSC-EVs suspensions contained them since all of them show a wide variety of phosphate- and carbohydrate-related stretches [62,63,64,65,66].

Raman spectroscopy, as a non-damaging, label-free, automated, sensitive, rapid, and non-destructive technique, allows us to implement biomarkers or specific proteins [68]; it was applied in this study to complement the characterization of the three types of EVs, to qualitatively supplement their chemical composition. The three biochemical fingerprints of the EVs were compared to each other to see if there was a significant difference. The results obtained reaffirm the ability of Raman spectroscopy to identify the presence of the main biochemical components of the EVs. The analysis of the spectra obtained from the MSC-EVs revealed that proteins and lipids contributed substantially to the Raman signals, which varied from one type of EV to another, since a higher signal was observed in the hDP-MSC-EVs for proteins, lipids, and nucleic acids; on the other hand, hE-MSC-EVs and hP-MSC-EVs also showed a response in the signal ranges of proteins, lipids, and nucleic acids, although with little significant difference concerning hDP-MSC-EVs. It should be noted that lipids have been shown to play a fundamental role in the biogenesis, stability, and fulfillment of the signaling functions of EVs, as well as being essential in the interaction between these and the recipient cells [68]. On the other hand, the strong presence of lipids in the bilayer of EVs can increase their rigidity and reinforce their morphology, allowing them to recover their integrity more easily when subjected to mechanical drug-loading methods. It has also been shown that EVs with a high partial lipid load improve the absorption or loading capacity of drugs, proteins, or nucleic acids, thereby increasing their efficacy in certain treatments [87,88,89,90].

To date, there have been studies on the complexity of the content of EVs using Raman spectroscopy to compare the chemical content of one source of EVs with another, as well as to evaluate the quality of isolation using different techniques [91]; in the present study, we focused only on the qualitative evaluation of the biochemical content of MSC-EVs, but significant differences were observed regarding the presence of certain molecules, which would imply an important role in their future application.

Based on the results of this study, it can be hypothesized that hDP-MSC-EVs could turn out to be the best candidates for their application as drug-loaded carriers since their proteomic profile indicates that they are derived from healthy cells with biologically active properties shared by their progenitor cells. On the other hand, they showed a larger size, which would imply a greater drug load due to a high surface area; a high zeta potential gives them greater stability, which could ensure good interaction with the drug and the medium. In general, they also showed a greater presence of lipids, proteins, and nucleic acids, which is an advantage if they are to be functionalized as vehicles for drugs or specific molecules; this is mainly due to the concentration of lipids in the membrane, since several biological actions depend on it, such as their stability, their capacity to encapsulate the drug in question, and their interaction with the target cell, since the lipid composition can influence how EVs fuse with cell membranes to release their contents, not to mention the biocompatibility and immunological tolerance that they generate. Although hP-MSC-EVs and hE-MSC-EVs showed good morphological and biochemical characteristics, compared to hDP-MSC-EVs, they showed smaller size and lower lipid and proteomic composition, which possibly makes them less capable of absorbing or loading drugs inside them and therefore, they generate a lower pharmacological response based on this principle; however, they are still excellent candidates as adjuvants for the treatment of diseases, since they share good similarities with their progenitor cells.

It is important to mention that a limitation in this type of study is the sample quality because an adequate isolation protocol is required to ensure a sample with a sufficient concentration of EVs, and their appropriate characterization, which facilitates their selection for specific applications. It is expected that future analyses in this field will be carried out with more concentrated and pure samples and, on the other hand, they can provide more information by applying and contrasting the results obtained by different methodologies.

In conclusion, the information obtained in this research is a guideline to generate new comparative analyses that facilitate the selection and application of MSC-EVs through the proposed methods and the free handling of the processing of EV samples, since the integrity of the EVs is taken care of, making the results of the analyses more reliable.

## 4. Materials and Methods

### 4.1. MSC-EV Suspensions

**Figure 7:**
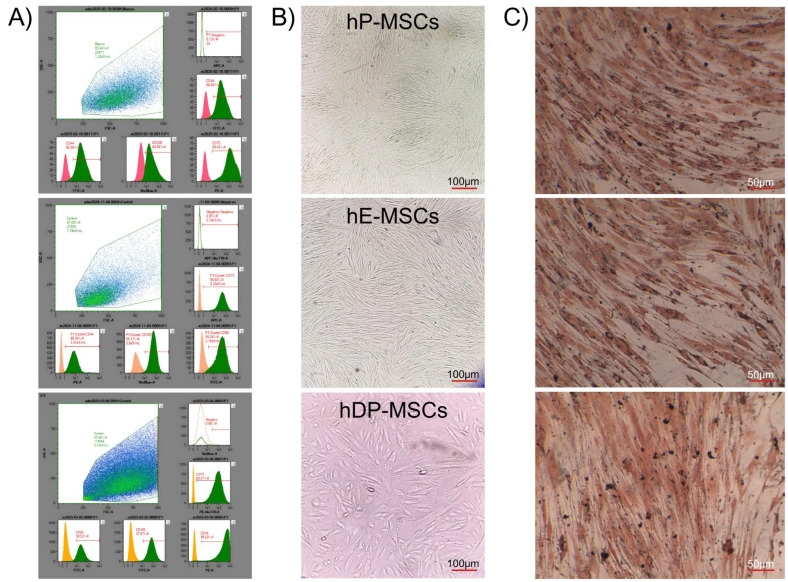
Isolation and identification of hP-MSCs, hE-MSCs and hDP-MSCs. (**A**) Markers (CD44, CD73, CD90, and CD105) detected by flow cytometry. (**B**) Micrography of adherent MSCs observed under a microscope. (**C**) Multipotency capacity of MSCs evaluated by Oil Red O staining.

MSC-EV suspensions (400 μg/7 mL) were provided by SANTER Biotechnology Center. Dental pulp-derived mesenchymal stem cells (hDP-MSCs) were isolated from the third molars of healthy young donors. Human endometrium-derived mesenchymal stem cells (hE-MSCs) were isolated from menstrual blood. Human placenta-derived mesenchymal stem cells (hDP-MSCs) were obtained from human placenta tissue which was collected from pregnant healthy women who underwent a cesarean section and were negative for HIV-I, hepatitis B, and hepatitis C. All samples were obtained after written informed consent. All MSCs were isolated and cultured according to previously described procedures [92,93,94]. The MSCs Phenotyping Kit, human (supplied by Miltenyi Biotec, Almac House, UK), was applied according to the manufacturer’s instructions to identify the cell surface markers of MSCs. The cells obtained from the three sources expressed markers such as CD44, CD73, CD90, and CD105, while lacking expression of CD14, CD19, CD34, CD45, and HLA-DR. These adherent cells also displayed the characteristic fibroblast-like spindle-shaped morphology of MSCs. In addition, the cells demonstrated multipotency, as they were able to differentiate into adipogenic, chondrogenic, and osteogenic lineages, as confirmed by microscopic analysis and staining quantification (Figure 7). Once MSCs reached 80% confluence, they were washed with PBS and then cultured in serum-free medium for 24 h. Then, MSC-EVs were isolated from the cell culture supernatant of the conditional media (CM) by a typical differential ultracentrifugation method [95,96]. Briefly, the CM underwent a low-speed centrifugation step (300× *g* at 4 °C for 10 min) followed by a higher-speed centrifugation (10,000× *g* at 4 °C for 30 min) to remove larger particles and debris. The samples were then filtered through a syringe or bottle-top filters with cellulose acetate membranes (0.22 µm pore size) to eliminate any remaining larger particles. To purify EVs using differential ultracentrifugation, the CM samples were first subjected to ultracentrifugation at 100,000× *g* at 4 °C for 70 min to pellet the EVs. A second washing step was performed by resuspending the EV pellet in PBS and conducting another 70 min of ultracentrifugation at 100,000× *g* at 4 °C. The EVs suspensions were stored at −80 °C until use.

### 4.2. Characterization of hP-MSC-EVs, hE-MSC-EVs and hDP-MSC-EVs

#### 4.2.1. Dynamic Light Scattering

The zeta potential and size distribution of EVs were analyzed by DLS in a Malvern Zetasizer 5000 instrument (Malvern Panalytical, Malvern, UK). All samples were diluted in 0.45 μm filtered double-distilled water (ddH_2_O) to an appropriate concentration before analysis. Each EV sample was measured three times. Between each sample measurement, the instrument chamber was washed with ddH_2_O.

#### 4.2.2. Transmission Electron Microscopy

TEM was used to visualize the morphology of EVs. Samples of each purified EV suspension were diluted in 0.45 μm filtered ddH_2_O to an appropriate concentration. Then, 6 μL of a dilute EV sample was pipetted onto a carbon-coated copper grid. Next, the grid was incubated at room temperature for 20 min. Excess liquid was removed by blotting. The dried grids were observed directly using a JEOL JEM 1010 electron microscope (JEOL Ltd., Akishima, Tokyo, Japan) operating at an accelerating voltage of 80 kV.

#### 4.2.3. Scanning Electron Microscopy

EV preparations were processed for scanning electron microscopy by resuspending in 0.1 μm filtered phosphate-buffered saline, fixed in 2% paraformaldehyde, and serially diluted in distilled water. For electron microscopy imaging, the samples were coated with a 2–5 nm layer of gold–palladium by sputtering to make their surfaces conductive. For observation, a JEOL JCM-6000plus benchtop SEM was used with an accelerating voltage of 15 kV.

#### 4.2.4. MACSPlex Surface Protein Profiling

**Figure 8:**
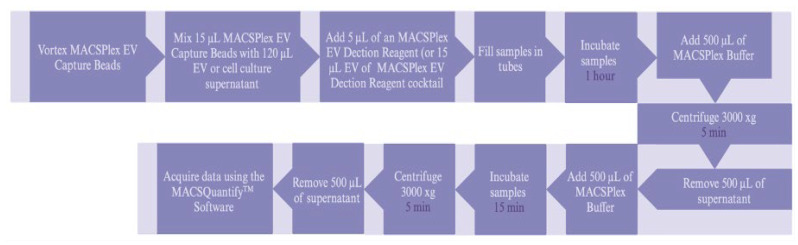
Experimental overview of the short tube protocol (drawn using the BioRender.com).

The MACSPlex EV IO kit is a bead-based flow cytometric kit used widely to analyze EV surface proteins. This kit can detect 37 surface epitopes (CD1c, CD2, CD3, CD4, CD8, CD9, CD11c, CD14, CD19, CD20, CD24, CD25, CD29, CD31, CD40, CD41b, CD42a, CD44, CD45, CD49e, CD56, CD62p, CD63, CD69, CD81, CD86, CD105, CD133.1, CD142, CD146, CD209, CD326, HLA-ABC, HLA-DR DP DQ, MCSP, ROR1, and SSEA-4), that are known to be present on different EVs, including tetraspanins CD9, CD63, and CD81, which are often referred to as common EV surface markers, plus two isotype control beads (mIgG1 and REA control). This protein profiling was performed employing the MACSPlex EV IO kit and a MACSQuant Miltenyi Biotec analyzer (Miltenyi Biotec Ltd., Woking, UK), and according to the manufacturer’s instructions (Figure 8).

The background values of phosphate-buffered saline and isotype controls (REA or mouse IgG) were subtracted from the sample’s median fluorescence intensity. The channels were compensated with the fluorochromes of the antibodies CD9, CD63, and CD81. The selection of the analysis population was carried out, with respect to the size (FSC) and granularity (SSC) graph, with the positive control population, the populations selected for the analysis, and the negative population.

#### 4.2.5. Fourier-Transform Infrared

FTIR spectroscopy was performed to analyze if there exists a difference in the chemical profile of each EV source. FTIR measurements were performed with a Nicolet S50 FTIR. From each sample of purified EV suspension, a drop of approximately 2 μL was deposited onto a diamond window. Measurements were performed in the wavenumber range of 4000–400 cm^−1^ using a spectral resolution of 2 cm^−1^. Fifty scans per sample were performed. ddH_2_O was also measured in triplicate as a control experiment. To analyze the secondary structure of proteins and other components possibly present in the EVs, the second derivative was applied to the FTIR data by JMP Pro 18.0.2.

#### 4.2.6. Raman Spectroscopy

For the Raman measurements, 500 μL of each MSC-EV sample was directly placed on a borosilicate glass tube. Next, the glass tube was put into a sampling accessory. The DXR2 SmartRaman Spectrometer (Thermo Scientific, Madison, WI, USA) with a 180-degree accessory was used to obtain the EV spectral collection. A 785 nm HP laser was equipped with a Raman spectrometer with an aperture of 50 um. The spectral range 700 cm^−1^ to 1700 cm^−1^ (left) and 2850 cm^−1^ to 3200 cm^−1^ (right) were implemented for the spectral collections. The exposure time was 25 s for 15 pulsations (375 in total per sample) at 100% laser power (150 mW). Spectroscopic measurements, including laser and Raman intensity calibrations, were fully automated by the OMNIC software version 9.2.0 using standard references located in the alignment/calibration module. To improve Raman fingerprinting of spectra, a second derivative was applied to data obtained with JMP Pro 18.0.2.

## Figures and Tables

**Table 1:** Known special features of EV subtypes.

	Exosomes	Ectosomes	Apoptotic Bodies
Size	30–150 nm	100–1000 nm	50–5000 nm
Biogenesis	MVBs	Plasma membrane	Cellular disassembly/fragmentation of cell components
Density	≈1.10–1.14 g/mL	≈1.12–1.20 g/mL	1.16–1.28 g/mL
Enriched proteins	Alix, TSG101, CD63, CD9, CD81, Syntenin-1	MMP2, CK18, Annexin A1, Annexin A2, CD147, CD9	Annexin V
Composition	Nucleic acids, proteins, lipids and metabolites.	Nucleic acids, proteins, lipids and metabolites.	Chromatin remnants, cytosol portions, DNA fragments and histone, degraded proteins.

Note: This table was adapted from the information published by [3,12,13,14,15,16,17,18]. Abbreviations: MVBs, multivesicular bodies; TSG101, tumor susceptibility gene 101 protein; CD9, CD63, CD81, tetraspanin proteins; CK18, cytokeratin 18; DNA, deoxyribonucleic acid.

**Table 2:** Therapeutic benefits of MSC-EVs.

MSC-EVs Source	Biomarker Characterization of MSC-EVs	Molecular Effect of MSC-EVs	References
Human adipocyte-derived MSCs	Alix, Hsp70, Flotillin-2 and CD9, as well as TGFβ	MSC-EVs reach the hippocampus, reduce glial activation and neuroinflammation, and restore cognitive function.	[38]
Human placenta-derived MSCs	CD9, CD63	MSC-EVs promote the tube formation and migration of human umbilical vein endothelial cells.	[39]
Human placenta-derived MSCs	CD63, CD81, CD9, and TSG101	MSC-EVs showed therapeutic effects on liver fibrosis.	[40]
Dental pulp-derived MSCs	HSP70, TSG101, CD9, CD63, CD81	MSC-EVs facilitated the transformation of pro-inflammatory macrophages into a pro-resolving phenotype, as demonstrated by elevated expression of M2 markers and reduced secretion of pro-inflammatory cytokines.	[41]
Dental pulp-derived MSCs	CD9, CD63, TSG101 and CD81	MSC-EVs significantly enhanced the expression of osteogenic transcription factors and enzymes, including runt-related transcription factor 2 and alkaline phosphatase, thereby increasing the osteogenic differentiation capacity of Hertwig’s epithelial root sheath cells.	[42]
Human endometrium-derived MSCs	CD63	MSC-EV MicroRNA-21 is a potential mediator of human endometrium-derived MSCs in therapy by enhancing cell survival through the PTEN/Akt pathway.	[43]
Human endometrium-derived MSCs	CD9 and CD63	MSC-EVs exert an antioxidant effect and increase the developmental competence of in vitro fertilization-derived embryos from older females.	[44]
Human bone marrow-derived MSCs	CD9 and CD63	MSC-EVs promoted cartilage regeneration in vitro, inhibited the adverse effects of inflammatory mediators on cartilage homeostasis, and stimulated the production of proteoglycans and type II collagen by these cells.	[45]
Bone marrow-derived MSCs	CD9 and CD63	MSC-EVs accelerated diabetic wound healing via enhanced angiogenesis.	[46]

Abbreviations: MSCs, mesenchymal stem cells; MSC-EVs, MSC-derived EVs.

**Table 3:** Surface markers identified in MSC-EVs and their functionality.

Surface Marker Antibodies (SMAs)	Target	SMAs Present in hP-MSC-EVs	SMAs Present in hE-MSC-EVs	SMAs Present in hDP-MSC-EVs	Reference
CD9, CD63, CD81	Tetraspanins, cell adhesion, migration, and signaling.	CD9, CD63	CD9, CD63	CD9, CD63	[47,48,49,50,51]
CD31, CD40, CD44, CD69, CD146, CD142	Cell origin (endothelial, epithelial and MSCs), and cell activation and EV functional status markers.	CD31, CD44, CD40, CD142	CD31, CD44, CD40, CD146, CD31, CD142	CD31, CD146, CD142	[47,52,53,54,55]
CD29, CD49e	Integrins, cell activation and EV functional status markers.	CD29	CD29, CD49e	CD29, CD49e	[52]
CD8, CD14, CD19, CD24, CD41b, CD42a, CD62P, CD86, R0R1, HLA-ABC	Lineage-associated markers present in healthy cells.	CD8 *, CD19 *, CD62P, R0R1 *	CD62P, CD86, CD41b *, HLA-ABC *	CD42a *, CD86	[47,52,56]
CD326	Epithelial cell adhesion molecule/EV functional status markers.	CD326	CD326	CD326	[48,52]
CD4, CD20, CD209	Markers associated with immune and antigen-presenting cells.	CD4 *, CD209 *	CD20	CD20	[48,56,57]
CD45	Hematopoietic marker.	CD45 *	N/A	N/A	[48,56,57]
CD133/1 (prominin-1)	Transmembrane protein present in MSCs.	CD133/1	CD133/1	CD133/1	[58,59]
HLA-DR	Negative cellular marker for MSCs.	N/A	N/A	N/A	[60]
MSCP	A type I transmembrane proteoglycan found on the cell surface that plays a crucial role in cell survival, migration, and the formation of new blood vessels.	MSCP	MSCP	MSCP	[61]

Note: Only those surface markers with APC fluorescence intensity above the mean were considered to be present. The * indicates that the marker was specific for the MSC-EV type. Abbreviations: MSCs, mesenchymal stem cells; EVs, extracellular vesicles; hP-MSC-EVs, EVs of human placenta-derived mesenchymal stem cells; hE-MSC-EVs, EVs of human endometrium-derived mesenchymal stem cells; hDP-MSC-EVs, EVs of human dental pulp-derived mesenchymal stem cells; N/A, Not Identified.

**Table 4:** Assignments of prominent peaks observed in EV IR spectra.

Wavenumber (cm^−1^)	Vibrational Mode	References
1300−1000 cm^−1^	Phosphates and carbohydrates	[62,63]
1700−1500 cm^−1^	Amide bands	[62,64]
1600−1700 cm^−1^	Amide I	[62,64]
1550, 1546, 1540, 1537 cm^−1^	Amide II	[62,64]
1743, 1728 cm^−1^	Interfacial region	[62]
1740 cm^−1^	Ester carbonyl	[63,65]
3100, 3078 cm^−1^	Amide B	[62,66]
3300, 3298, 3290, 3285 cm^−1^	Amide A	[62,66]

**Table 5:** Raman shift values and tentative band assignments of the Raman spectrum.

Raman Shift (cm^−1^)	Tentative Assignment	References
700–704	Lipids	[67]
725–751	Nucleic acids	[67]
770–776	Nucleic acids	[68]
812–839	Nucleic acids, phospholipids	[67,68]
838	Tyr	[69]
852–859	Pro: *ν*(C-C), Tyr: ring breathing (proteins), *polysaccharide structure*	[67,68,69]
886	*Phospholipids*	[67]
900	*Phospholipids*	[67]
934–935	*C-C backbone*/*C-C stretching* (*collagen*/*protein assignment*)	[67,68,70]
928–960	*Polysaccharide structure*, *d(C-C-N) symm*, *a-helical skeletal*, *proteins*	[67,68,69]
971	*p(CH2)* (*lipids*)	[71,72]
1000–1008	Proteins	[70]
1003	*Phenylalanine*	[68]
1000–1008	Phe: ring breathing (proteins)	[70]
1090–1100	Nucleic acids	[67]
1050–1160	Proteins	[67]
1155–1160	Carotenoids	[67]
1169–1180	Nucleic acids and proteins	[70]
1552–1554	*Tryptophan* (*protein assignment*)/*porphyrin*/*amide II*	[73]
1200–1208	Nucleic acids and proteins	[67,70]
1200–1260	Nucleic acids	[67]
1295–1314	Lipids	[67,70]
1355–1365	Nucleic acids and proteins	[70]
1400–1430	Proteins	[70]
1420–1470	Lipids	[70]
1515–1540	Carotenoids	[67,70]
1570–1580	Nucleic acids	[67]
1602–1604	Lipids	[67]
1619–1622	Proteins	[67,70]
1640–1700	Nucleic acids, lipids, and proteins	[67,70]
1720–1750	Lipids	[67]
2928	Lipids	[70]

## Data Availability

Data are contained within the article.

## Abbreviations

EVs	Extracellular vesicles
MSCs	Mesenchymal stem cells
MSC-EVs	Extracellular vesicles derived from mesenchymal stem cells
MVs	Microvesicles
MVBs	Multivesicular bodies
hP-MSCs	Human placenta-derived mesenchymal stem cells
hE-MSCs	Human endometrium-derived mesenchymal stem cells
hDP-MSCs	Human dental pulp-derived mesenchymal stem cells
hP-MSC-EVs	EVs of human placenta-derived mesenchymal stem cells
hE-MSC-EVs	EVs of human endometrium-derived mesenchymal stem cells
hDP-MSC-EVs	EVs of human dental pulp-derived mesenchymal stem cells
DLS	Dynamic light scattering
CM	Conditional media
SMAs	Surface marker antibodies
PDI	Polydispersity index
TEM	Transmission electron microscopy
SEM	Scanning electron microscopy
FTIR	Fourier-transform infrared spectroscopy
ddH_2_O	Filtered double-distilled water
